# Characterizing the activity of abundant, diverse and active CRISPR-Cas systems in lactobacilli

**DOI:** 10.1038/s41598-018-29746-3

**Published:** 2018-08-01

**Authors:** Alexandra B. Crawley, Emily D. Henriksen, Emily Stout, Katelyn Brandt, Rodolphe Barrangou

**Affiliations:** 1 North Carolina State University Functional Genomics, Raleigh, NC 27695 USA; 2 North Carolina State University Department of Food, Bioprocessing and Nutrition Sciences, Raleigh, NC 27695 USA

## Abstract

CRISPR-Cas systems provide immunity against phages and plasmids in bacteria and archaea. Despite the popularity of CRISPR-Cas9 based genome editing, few endogenous systems have been characterized to date. Here, we sampled 1,262 publically available lactobacilli genomes found them to be enriched with CRISPR-Cas adaptive immunity. While CRISPR-Cas is ubiquitous in some *Lactobacillus* species, CRISPR-Cas content varies at the strain level in most *Lactobacillus* species. We identified that Type II is the most abundant type across the genus, with II-A being the most dominant sub-type. We found that many Type II-A systems are actively transcribed, and encode spacers that efficiently provide resistance against plasmid uptake. Analysis of various CRISPR transcripts revealed that guide sequences are highly diverse in terms of crRNA and tracrRNA length and structure. Interference assays revealed highly diverse target PAM sequences. Lastly, we show that these systems can be readily repurposed for self-targeting by expressing an engineered single guide RNA. Our results reveal that Type II-A systems in lactobacilli are naturally active in their native host in terms of expression and efficiently targeting invasive and genomic DNA. Together, these systems increase the possible Cas9 targeting space and provide multiplexing potential in native hosts and heterologous genome editing purpose.

## Introduction

CRISPR-Cas (Clustered regularly interspaced short palindromic repeats and CRISPR associated genes) systems have been shown to protect bacteria and archaea from invasive mobile genetic elements (MGEs)^1–3^. These systems are identified by a genetic locus with a CRISPR repeat-spacer array and *cas* genes^4,5^. During *adaptation*, the first stage of CRISPR immunity, foreign DNA sequences from MGEs are copied and pasted iteratively into the array as unique spacer sequences flanked by conserved repeats^4,6–9^. The second stage of CRISPR immunity, *expression*, leads to the biogenesis of individual small crRNAs (CRISPR RNAs), that each contain a single partial spacer and partial repeat; these RNAs act as a guide molecule to direct the Cas proteins to a complementary foreign nucleic acid target^2,10,11^. Some specific subtypes of CRISPR-Cas systems, including Type II-A, require a second RNA molecule, called the tracrRNA (trans-acting CRISPR RNA), to generate the individual crRNAs capable of guiding the signature Cas9 endonuclease^11–13^. The final stage of CRISPR immunity, *interference*, is the targeting and cleavage of foreign DNA when it is reintroduced into the cell^4,11,14^. Cas proteins are able to distinguish self from non-self targets through the occurrence of a PAM (protospacer adjacent motif) on the foreign target that is not present when then spacer is stored in the repeat-spacer array^7,15–17^.

CRISPR is fairly common in bacteria, occurring in just under half of all bacterial species sequenced to date in publically available databases^18,19^. Though the stages of CRISPR-Cas immunity are universal, there are two main classes of systems that can be further broken down into six types and 23 subtypes that utilize different Cas proteins and crRNA structures^19–21^. Though Type II-A systems can only be identified in 5% of bacteria, they are arguably the most used, since the molecular machinery from this subtype can be repurposed to generate Cas9-based genome editing tools^4,5,22–24^. Despite being relatively rare, Type II-A systems are known to occur preferentially in firmicutes, like lactic acid bacteria, occurring in almost 30% of all lactobacilli^19,25,26^.

Interestingly, the majority of our knowledge of CRISPR activity in their native host has been limited to a few model systems, namely *Streptococcus pyogenes* (Type II-A)^23^, *Streptococcus thermophilus* (two Type II-As, one Type I-E, and one III-A)^1,6,11,13–15,27,28^, *Sulfolobus islandicus* (Type III-B)^29,30^, *Pseudomonas aeruginosa* (Type IE and IIA)^31,32^, and *Escherichia coli* (Type I-E)^2,8,33^. Unfortunately, some CRISPR systems in *E*. *coli* and other organisms do not appear to be natively active and most work must be performed *in vitro* or with heterologous CRISPR machinery, leaving our knowledge of native activity in the original somewhat shallow.

With relatively little known about the native activity of many different endogenous systems, we first identified a large selection of uncharacterized CRISPR-Cas systems. To fully characterize the Type II systems, we then predicted all system components for each system, including the PAM, tracrRNA, and crRNA. Next, we determined CRISPR interference to assess whether each individual system was active through investigating acquisition, expression, and interference. Finally, we used one model system, *Lactobacillus gasseri*, to investigate a novel species of tracrRNA to develop biotechnological CRISPR-Cas9 based genetic engineering tools using the native CRISPR components.

## Results

### Lactobacilli encode complete, diverse, and active CRISPR-Cas systems

**Figure 1:**
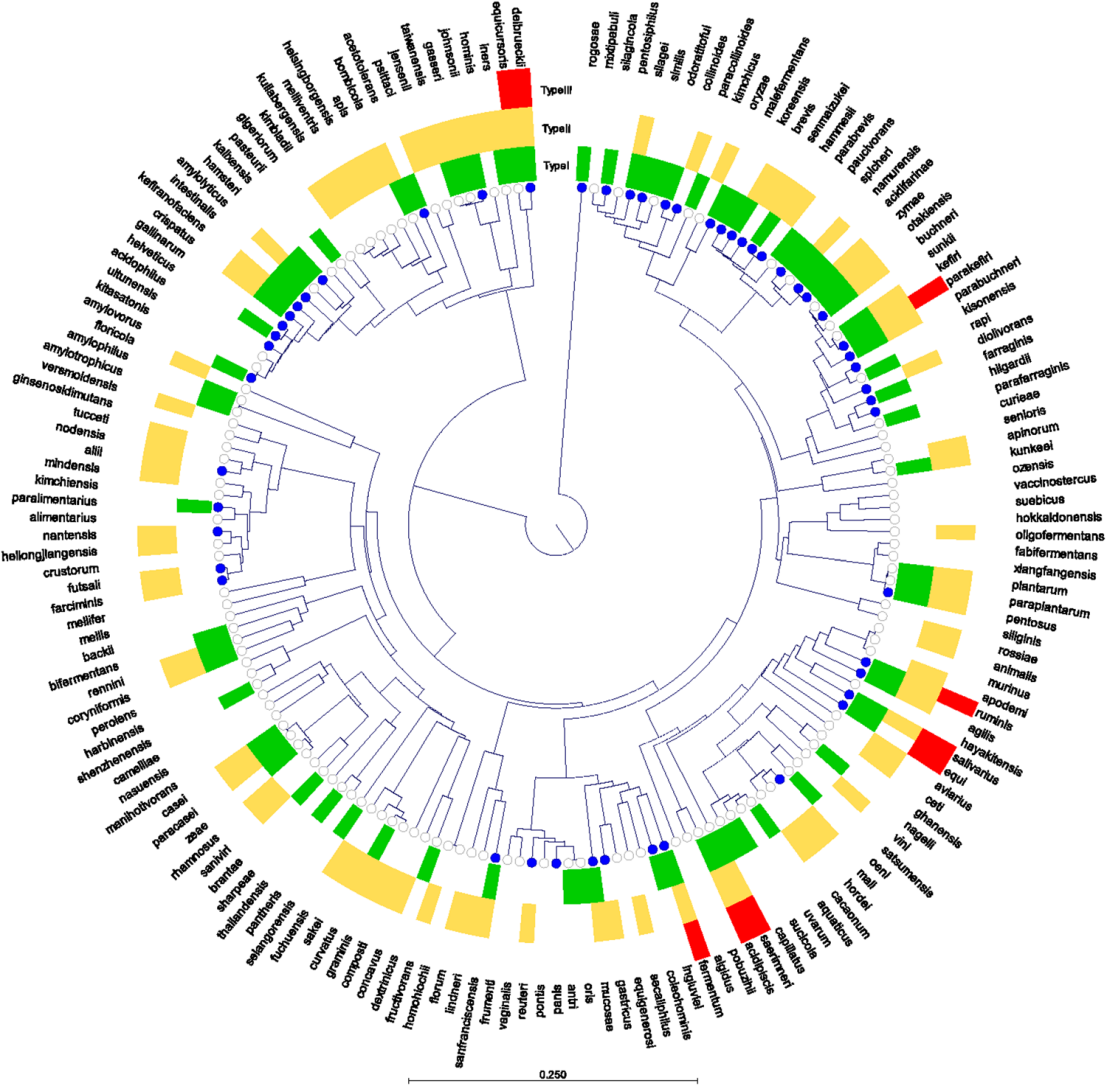
Occurrence of CRISPR-Cas systems in lactobacilli. The core genome of lactobacilli was identified by Sun *et al*. 2015. This tree displays the phylogenetic relationship of one representative genome from each of the 171 lactobacilli species used in this study based on the core genome. The metadata rings display the presence of CRISPR-Cas systems in any strain from that species. Type I systems are shaded in green, Type II in yellow, and Type III in red. The nodes are colored blue if a Type V-U putative Cas protein was identified in that species. A lack of color demonstrates a lack of CRISPR-Cas systems. The species is listed in the outer ring.

**Figure 2:**
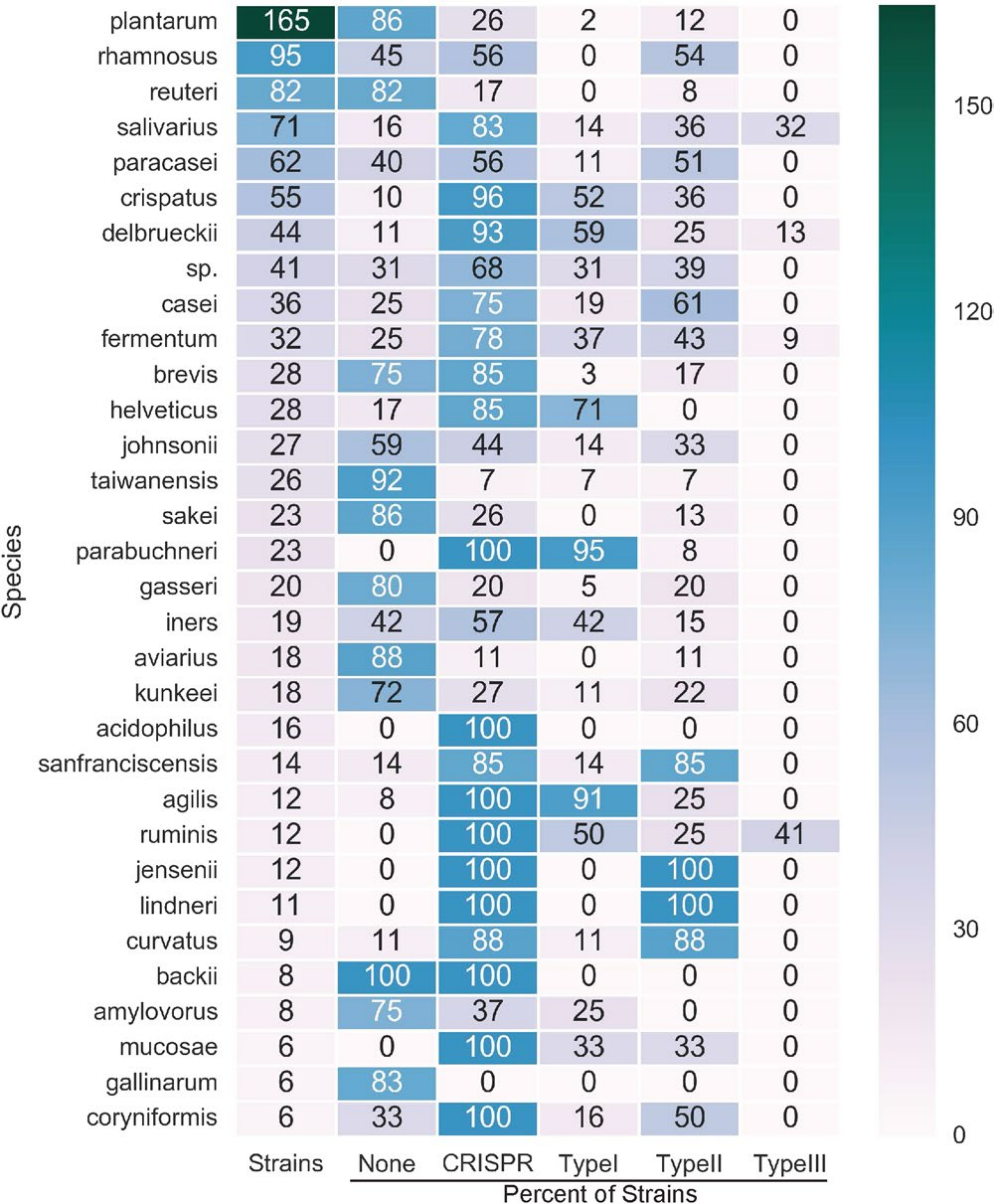
Strain-specific distribution in lactobacilli. For species where there were at least 6 representative genomes, the rate of occurrence of CRISPR repeats and complete systems is displayed. The number of strains investigated is in the first column. The remaining columns list the percent of strains containing: No *cas* genes, CRISPR repeats, Type I systems, Type II systems, or Type III systems.

**Figure 3:**
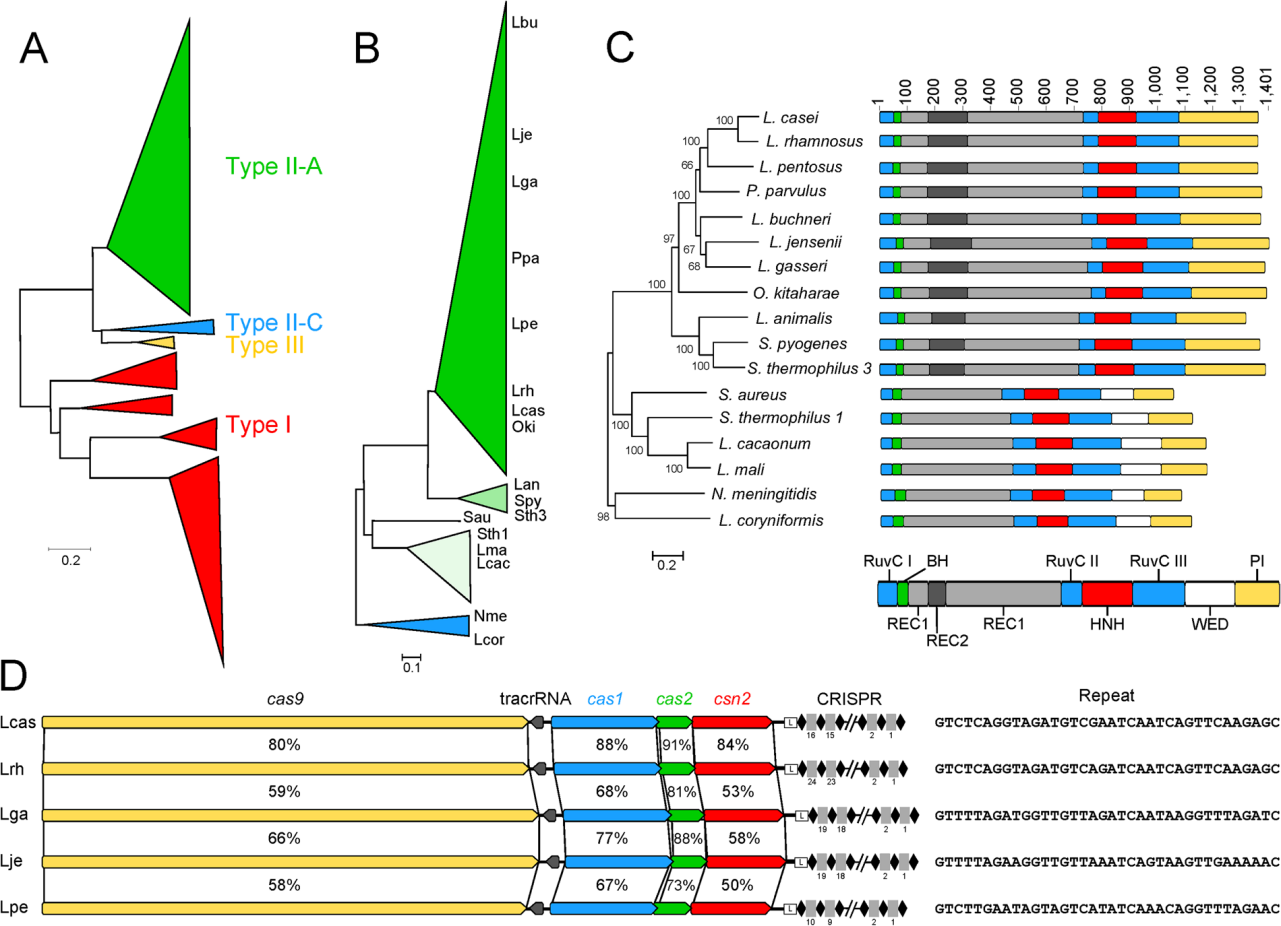
Diversity of CRISPR-Cas systems in lactobacilli. (**A**) The total diversity of CRISPR-Cas systems in lactobacilli was determined through the phylogenetic distribution of the Cas1 proteins. The ML tree is rooted on the Type I to Type II split. (**B**) Diversity of Type II systems was determined through alignment of the Cas9 protein. This tree is rooted on the outgroup II-C. (**C**) Cas9 protein domains were mapped from known protein crystal structures. The long II-A Cas9s – any Cas9 longer than 1250 amino acids – was mapped to the Streptococcus pyogenes Cas9. The short II-A and II-C Cas9s – less than 1,250 amino acids – were mapped to the Staphylococcus aureus Cas9. (**D**) Comparisons of entire CRISPR loci revealed high amounts of diversity in all Cas proteins (yellow arrow – Cas9, blue arrow – Cas1, green arrow - Cas2, red arrow - Csn2), tracrRNA sequence (dark grey arrow), leader sequence (box L), array length (black diamonds - repeats, grey rectangles - spacers), and CRISPR repeat sequence. The percent identities for the Cas proteins compare the protein above and below the percentage.

Despite the growing popularity of CRISPR-Cas, only a handful of systems have been characterized to date. We set out to understand the native variability in occurrence and activity of CRISPR using endogenous systems occurring in lactobacilli, as it has been published that they are enriched in CRISPR-Cas systems 6-fold compared to the canonical rate of occurrence for bacteria (5% of all bacteria vs. 30% of all lactobacilli)^19,25^. Our *in silico* searches of 1,262 strains of lactobacilli, accounting for 171 different *Lactobacillus* species and closely related lactic acid bacteria, confirmed diversity across both classes of systems, focusing on Types I, II and III (Figs 1, 2, 3 Panel A, Table S1). We were unable to detect Type IV, V or VI CRISPR-Cas systems in lactobacilli, though several V-U proteins were detected in our genomes (Table S1). Noteworthy, these results are consistent with previous studies documenting that Types I, II and III are most dominant and widespread in nature, though the size of the Type I arrays are smaller than the reported average array size for this type^34^. As these V-U systems are still putatively uncharacterized, we have not included them in determining the rate of occurrence of CRISPR-Cas systems in lactobacilli^20,35^.

We detected CRISPR repeats in 59.7% (753 of 1,262) of lactobacilli genomes and most often detected a single CRISPR-Cas locus in a genome (Fig. 2). Two strains of lactobacilli contained Type I, II and III systems in the same genome: *Lactobacillus fermentum* (strains NB-22 and MTCC 8711), *Lactobacillus equicursoris* (strain 66c) (Fig. 1). Multiple systems were often detected in the same genome; occasionally this corresponded to a single subtype with two distinct sets of *cas* gene and CRISPR arrays, but most often corresponded to a distinct Type I-E and II-A system in the same genome. The subtype I-E was the predominant Type I system identified in lactobacilli, accounting for 210 of the 268 Type I systems identified (Figs 1, 2). Likewise, the II-A subtype was the predominant Type II system, accounting for 290 of the 393 Type II systems identified. CRISPR-Cas systems are ubiquitous in 14 of the 171 species (*Lactobacillus parabuchneri*, *Lactobacillus jensenii*, *Lactobacillus ruminis*, *Lactobacillus agailis*, *Lactobacillus linderi*, *Lactobacillus mucosae*, *Lactobacillus pentosus*, *Lactobacillus farcimins*, *Lactobacillus kefiranofaciens*, *Lactobacillus animalis*, *Lactobacillus kefiri*, *Lactobacillus buchneri*, *Lactobacillus parakefiri*, *Lactobacillus equicursoris*) analyzed here and are rarely found in eight species (*Lactobacillus plantarum*, *Lactobacillus reuteri*, *Lactobacillus taiwanensis*, *Lactobacillus sakei*, *Lactobacillus gasseri*, *Lactobacillus avarius*, *Lactobacillus gallinarum*, *Lactobacillus paralimentarius*). There are three species that always contain CRISPR repeats but are always devoid of *cas* genes (*Lactobacillus acidophilus*, *Lactobacillus backii*, *Lactobacillus crustorum*); and conversely, one species, *Lactobacillus paracollinoides*, that always contains *cas* genes, but never contains CRISPR repeats.

The most notable CRISPR trend in lactobacilli is the enrichment of Type II systems, expanding the known Cas9 space to novel proteins, including short II-A Cas9s, long II-A Cas9s, and II-C Cas9s (Fig. 3). The Cas9s from lactobacilli contain an entire clade of Cas9s that is divergent from the canonical Cas9s, mainly *S*. *pyogenes* (Spy), *S*. *thermophilus* CRISPRs 1 and 3 (Sth1, Sth3, respectively*)*, *Staphylococcus aureus* (Sau), and *Neisseria meningitides* (Nme) (Fig. 3 Panel B)^1,12,14,36,37^. Though the lactobacilli Cas9 proteins contain the same motifs as the canonical Cas9s, they are highly dissimilar, sharing sometimes as low as 40% similarity at the protein coding level with Spy, Sth1, Sth3, Sau or Nme. Even within the clade of lactobacilli-specific Cas9s, there is great diversity in protein sequences, sometimes as low as 60% similarity to other lactobacilli Cas9s.

In CRISPR biology, *cas1* is currently considered the universal gene as it is found in most CRISPR-Cas systems and drives the acquisition stage of immunity^9,19^. Despite *cas1* being the universal *cas* gene, *cas2* was the most conserved gene amongst all *cas* genes identified (Fig. 3 Panel D). In addition to *cas* conservation and divergence, we observed evidence of maintenance and activity in the CRISPR arrays. The arrays contained between 2 and 135 spacers, with the median array containing 20 spacers. On average, the Type I systems contained the longest CRISPR arrays (27 spacers Type I, 19.5 spacers Type II, 9 spacers undefined) (Table S1). When arrays are inactivated, they can accumulate mutations in repeats and show evidence of degeneration through inconsistent length of repeats and spacers^14,26^; in contrast, CRISPR repeats in lactobacilli remain intact in terms of length and sequence across the entire array suggesting they are still actively maintained and functional.

### crRNA biogenesis and active transcription of CRISPR RNAs

**Figure 4:**
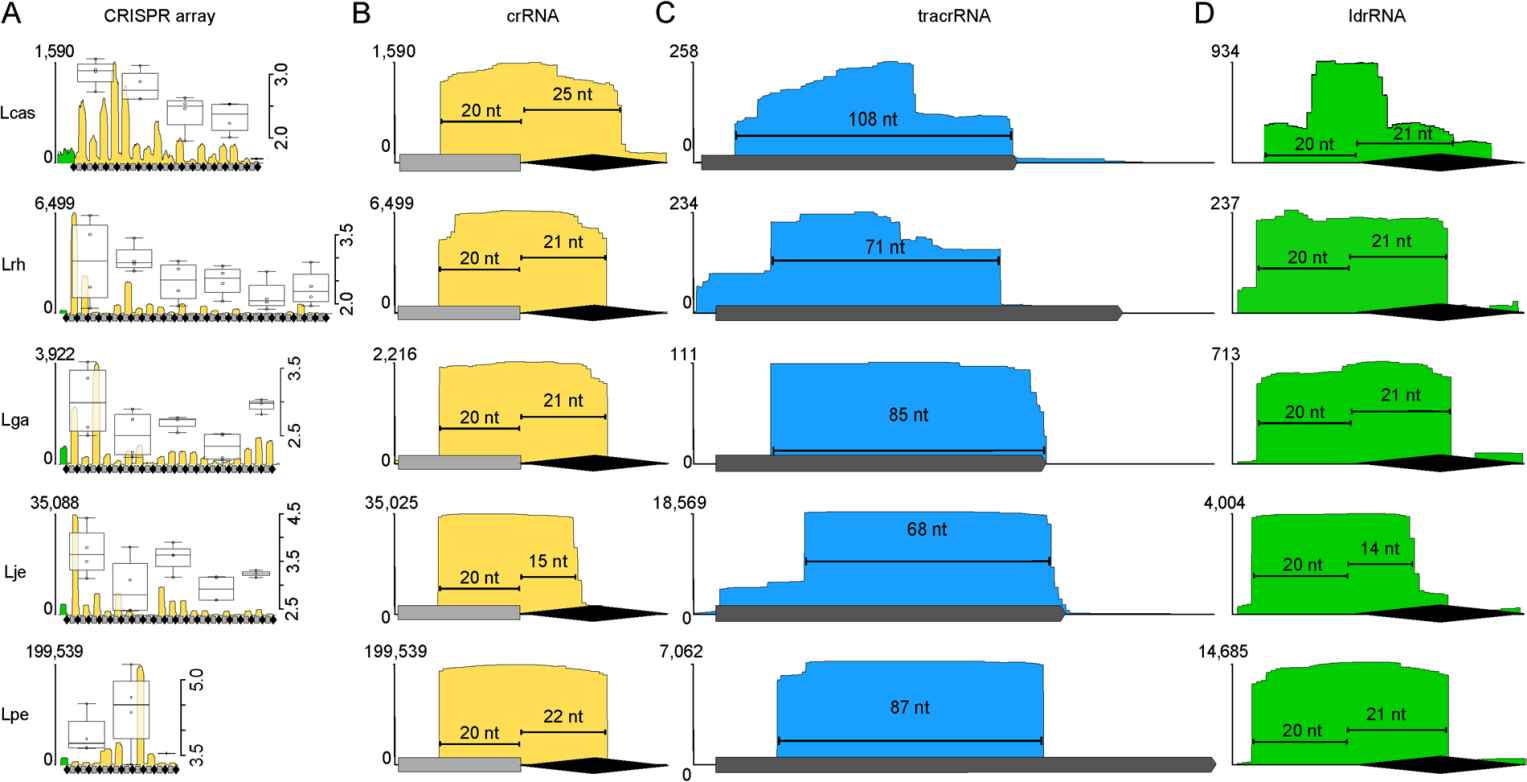
Expression of CRISPR transcripts. (**A**) Expression profile of the entire CRISPR-Cas array reveals the transcription levels of the ldrRNA (green) and crRNAs (yellow) for *L*. *casei* (Lcas), *L*. *rhamnosus* (Lrh), *L*. *gasseri* (Lga), *L*. *jensenii* (Lje), and *L*. *pentosus* (Lpe). The left y-axis shows the sequencing coverage depth at each position; the right y-axis shows the log transformed coverage depth for the box plots. Over laid box plots show the distribution of transcription level in four crRNA increments; the transcript level for each individual crRNA is marked by open circles in the box plots length (black diamonds - repeats, grey rectangles - spacers). (**B**) The boundaries of a highly expressed crRNA is shown for each organism. The length of the spacer portions of the crRNA (grey rectangle), is strictly conserved at 20 nt for all crRNAs. The length of the repeat portion of the crRNAs (black diamond), varies between organisms. (**C**) The tracrRNA transcriptional profile reveals the boundaries of tracrRNA processing. The gray bar on the x-axis is the *in silico* prediction for the tracrRNA. (**D**) The transcriptional profile for the ldrRNA for each organism closely matches the crRNA transcriptional profile.

Expression is the second stage of CRISPR interference. To determine the activity of CRISPR expression in lactobacilli, we investigated the crRNA transcripts via small RNA-Sequencing. We were able to determine that crRNAs were some of the most highly transcribed small RNAs in cells, even reaching 199,539 transcripts of a single crRNA in *Lactobacillus pentosus* (2.5%, in 8,000,000 total reads), making that crRNA the 4^th^ most highly expressed small RNA in the cell (Figs 4, S1, S2). When visualizing the crRNA transcripts, we found it very striking to observe the sharp boundaries of processed crRNAs; this demonstrates the cleavage of pre-crRNAs to individual crRNAs is precise and consistent. As seen with other organisms, the length of processed crRNAs was conserved within an array but differed between systems. Interestingly, the spacer portion of the crRNA was consistently 20 nucleotides long in all Type II-A crRNAs (Figs 4, S1, S2). Interestingly, the repeat portion of the crRNA was unique to each CRISPR system, ranging from 13 nucleotides in *Oenococcus kitaharae* to 25 nucleotides in *L*. *casei*. The II-C crRNAs in *Lactobacillus coryniformis* were comprised of 17 nucleotides in the spacer portion and 22 nucleotides in the repeat portion.

We observed an interesting trend in the expression pattern of the first repeat in the CRISPR array. The 5′ end of the leader RNA, ldrRNA, as we propose to name it, contains 20 nucleotides of the promoter-like leader sequence (Figs 4, S3). The length of the leader transcribed in the ldrRNA is the same length of spacer sequence transcribed in the downstream crRNAs and the length of the repeat transcribed in the ldrRNA is also the same length of repeat transcribed in crRNAs. This RNA was first seen in *S*. *thermophilus* by Wei *et al*.^28^, but this finding has not been investigated in other organisms.

**Figure 5:**
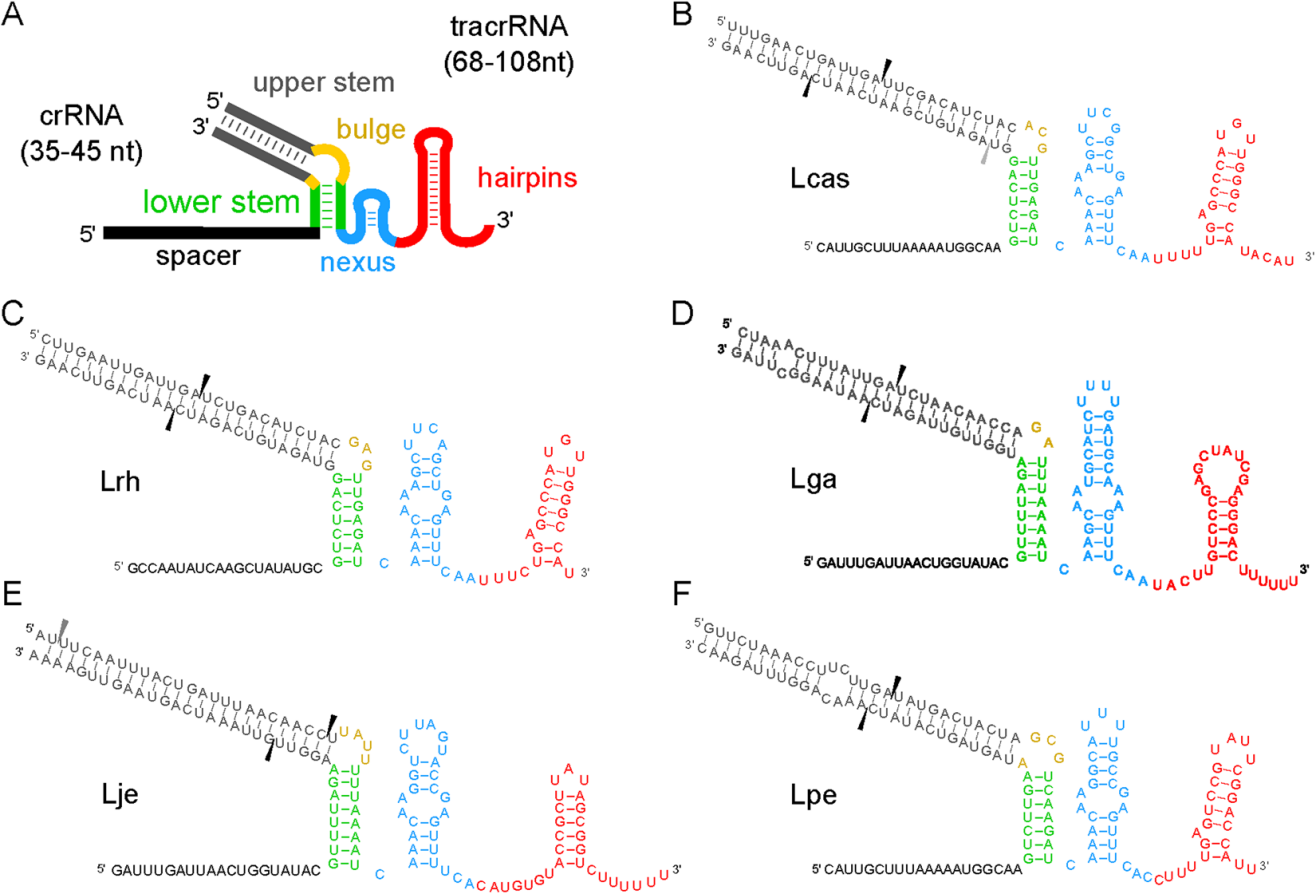
crRNA:tracrRNA duplexes. (**A**) The consensus structure of each crRNA:tracrRNA duplex is displayed in Panel A. Together, the crRNA:tracrRNA duplex forms the lower stem (green), bulge (yellow), and upper stem (dark grey) modules. The crRNA contains the spacer (black) module, and the tracrRNA contains the nexus (blue) and terminal hairpins (red). (**B**–**F**) The duplex for each organism is displayed with the RNase III processing sites (black rectangles) as determined by the transcriptional boundaries. Secondary processing sites when applicable are shown by grey arrows.

The tracrRNAs were predicted *in silico* according to Briner *et al*.^12^, looking for the 5 modules found in the tracrRNA: upper stem, bulge, lower stem, nexus and ending with one to three terminal hairpins; one of which being a GC-rich transcription terminating hairpin (Fig. 4). The expression boundaries of the tracrRNAs are clearly defined, further demonstrating the expression stage of CRISPR-Cas immunity is active. We found that our predictions for the tracrRNAs were often too conservative and the transcription terminating hairpins are often not a part of the final tracrRNAs (Figs 4, S4). As a consequence, in lactobacilli, there is most often only a single terminal hairpin, though two or three were typically predicted (Figs 5, S4, S5). The RNaseIII processing sites are best determined via boundary mapping, as they are often unpredictable^38,39^. All but two of the tracrRNAs we looked at contained the bulged stem loop nexus typical of and unique to lactobacilli. Among the tracrRNAs investigated here, five groups are completely unique and likely orthogonal to other systems known to date based on the predicted structures of the sgRNAs, the Cas9 sequences, and their predicted PAM targets.

### Interference stage is active against foreign DNA

The final stage of CRISPR interference is sequence-specific targeting and cleavage of complementary foreign DNA upon introduction to the cell. To determine whether the CRISPR systems were active, we first needed to determine what sequences these systems natively target. The protospacers corresponding to the spacer sequences already stored in CRISPR arrays revealed these systems provide immunity against phages, plasmids, and prophages (Fig. 5, Table S2). In particular, *L*. *jensenii* is under strong predatory pressure from phage LV-1 as 10 different spacers target separate sequences on the same phage (Table S2). Beyond predatory phages, many spacers targeted prophage and mobile elements such as transposons, suggesting that beyond immunity, CRISPR-Cas systems might be active in maintaining genome homeostasis and helping control the flow of horizontal gene transfer.

**Figure 6:**
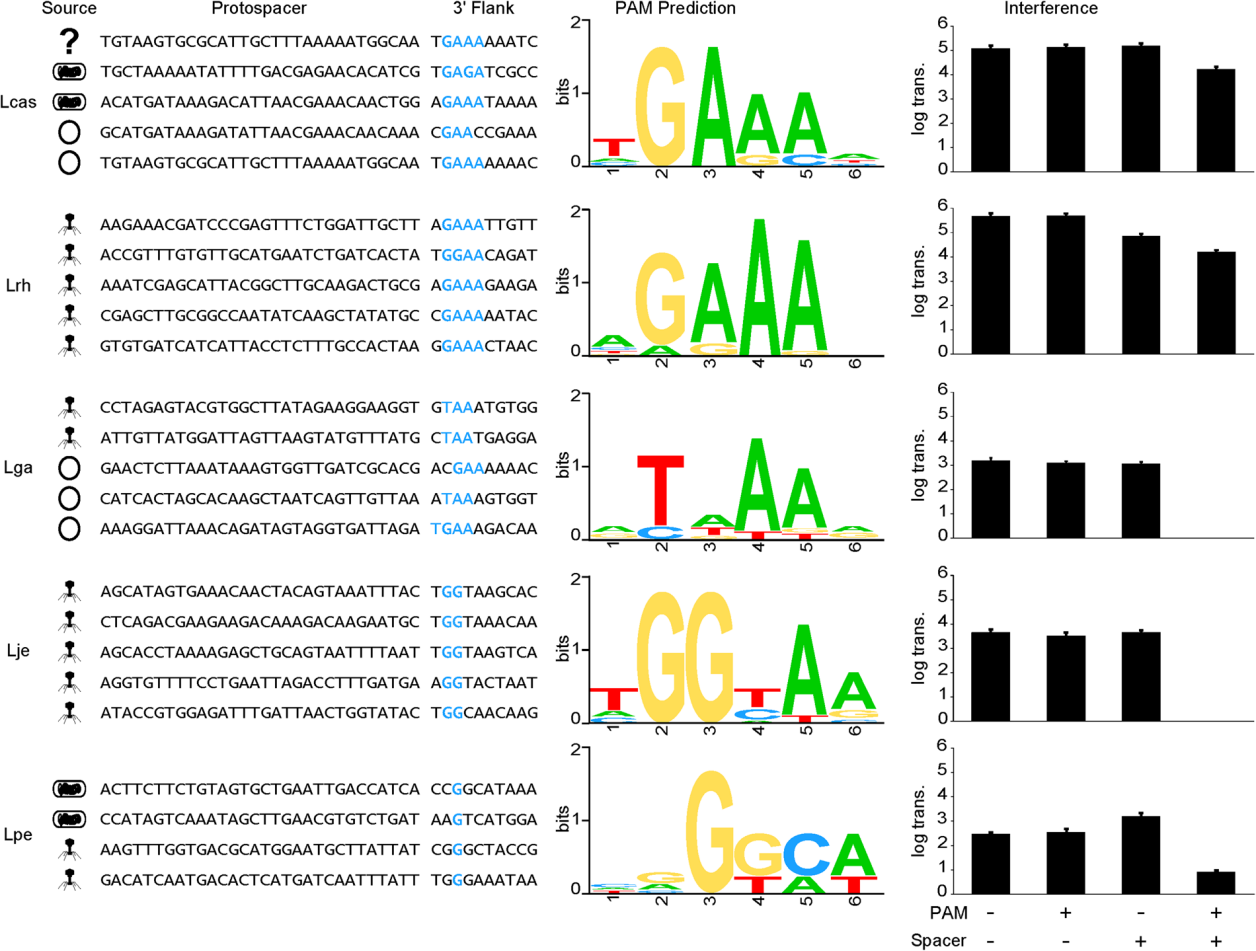
PAM prediction and validation. Representative spacers from each organism are displayed with their protospacer targets. The source of each protospacer sequence was determined to be either phage (phage), plasmid (circle), chromosomal origin (bacterial cell), or unknown metagenomics origin (?). Ten nucleotides from the 3′ flank for each protospacer was used to predict the PAM sequence (blue text) for each Cas9. All of the 3′ flanks for each protospacer were aligned to generate a Weblogo for each PAM prediction. Finally, plasmid interference assays were used to test the ability of each Cas9 to recognize and cleave plasmid DNA. Constructs containing PAMs and protospacers homologous to native spacers in the host genome were used to determine plasmid interference. The log number of transformants is plotted to show the efficiency of the native CRISPR system in eliminating each construct; error bars are based on three independent replicates. The most effective PAM for each organism is shown: Lcas 5′-tGAAAA-3′, Lrh 5′-aGAAA-3′, Lga 5′-cTAACc-3′, Lje 5′-tGGc-3′, and Lpe 5′-gTTAAT-3′.

The PAM sequences were predicted using the flanking regions of the protospacers. To test whether Cas9 was able to recognize these predicted PAMs, we cloned a native spacer sequence from each endogenous array into a plasmid and included the predicted PAM and tested several mutated variants. The plasmid interference assay was able to determine whether Cas9 is able to recognize the PAM sequence provided, and also demonstrated that the system was active through the ability of Cas9 to target and cleave the foreign DNA and preventing the uptake of plasmid DNA (Figs 6, S6).

We were able to demonstrate that five different CRISPR-Cas systems have endogenous interference activity, with a range of interference efficiencies. One phenomenon we observed was flexibility in PAM targeting by Cas9, which was seen most prominently in *L*. *gasseri* (Figs 6, S6). The PAM 5′-cTAAC-3′ performed perfect interference and did not have any escapees, while the PAM 5′-ccAAC-3′ allowed one log of transformants to survive and the PAM 5′-cTAAAC-3′ allowed two logs of transformants to survive. In prior experiments, the imperfect PAM 5′-nTAAAC-3′ was shown to allow some escapees^40^.

### Repurposing endogenous systems using self-targeting sgRNAs

**Figure 7:**
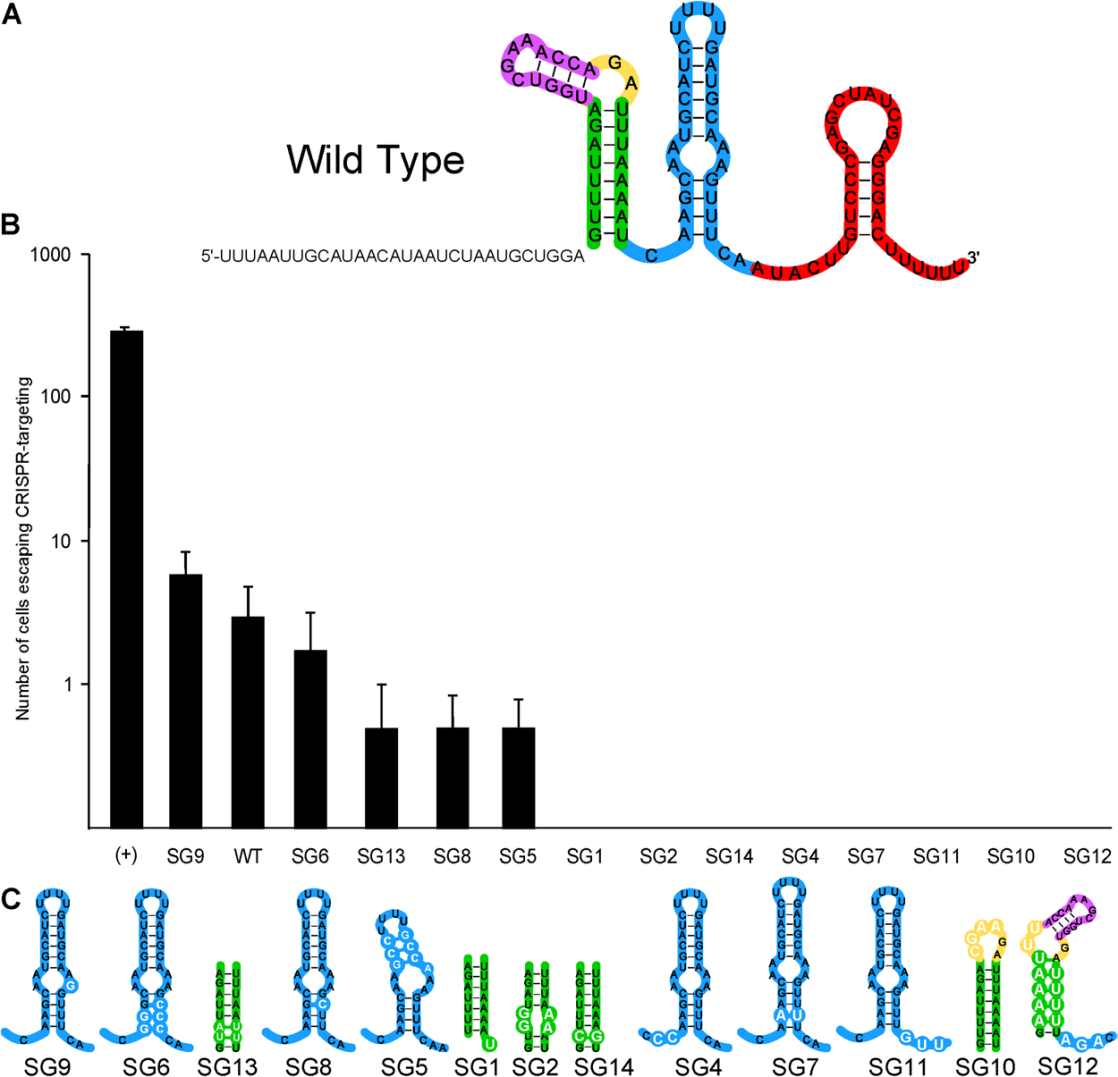
Self-targeting assays. (**A**) An Lga single guide RNA was designed to target the chromosome. (**B**) The ability of each guide to target and cleave the chromosome determines the transformation efficiency of each guide. Error bars are based on three independent replicates. (**C**) Mutations were made to particular modules in the nexus (blue module), the lower stem (green module), the bulge (yellow module) and the upper stem (purple module). Each construct is named SG for Single Guide. The wild type guide is called WT.

In addition to validating the systems’ ability to target invasive plasmid DNA, we also wanted to test whether we could co-opt native Cas9 machinery and turn the system into a self-targeting chromosomal DNA cleavage system. We designed sgRNAs based on the *L*. *gasseri* tracrRNA:crRNA duplex and cloned them into an expression vector using the native constitutive *tuf* promoter (Figs 7, S7). The wildtype sgRNA was able to reduce the number of transformants by 3 logs. Once we had validated the ability of the system to commandeer the native Cas9 and perform self-targeting cleavage and death, we wanted to determine which perturbations in the sgRNA would still permit Cas9 binding and utilization. As the guide perturbations from Briner *et al*. 2013 demonstrated that mutations to the nexus and terminal hairpin is often the most detrimental to Cas9 utilization, we focused on mutations to these modules. Interestingly, the adenine residues in the nexus bulge (SG9, Fig. 7) may interact in a sequence-specific manner with Cas9 as the single point mutations to those nucleotides reduced the interference activity of Cas9 by almost a full log of transformants. Surprisingly, we were able to increase the efficiency of Cas9 targeting by changing some of the AU-rich stem pairing to GC-rich pairing in the nexus and lowerstem (SG1,14, 4, 7, 11, 10, 12).

## Discussion

The phylogenetic distribution of novel CRISPR-Cas systems suggests there remains an uninvestigated diverse pool of CRISPR-Cas systems with potential different efficiencies, targeting PAMs, and guide RNAs structures. Here, we have set out to determine the diverse activities in several novel Type II CRISPR-Cas systems. The high level of divergence suggests these proteins are actively evolving, likely in response to selective pressure, possibly including phage inactivation using anti-CRISPR proteins^41–43^. The diversity seen in *cas1* suggests on-going evolution and thus the acquisition or adaptation stage of CRISPR might still be active in some lactobacilli, which is a rare event and has only been seen naturally in *S*. *thermophilus*^6,14,15,28^ and *Haloarcula hispanica*^44,45^ and artificially in *E*. *coli* and *S*. *aureus*^9,46–49^.

While the crRNAs investigated here match the canonical length for previously investigated Type II-A crRNAs in Spy and Sth^10,11,22^, the crRNAs in II-C CRISPR-Cas systems should be further investigated to determine if these lengths are conserved as they are in II-A systems. The length of the spacer portion of the crRNA is well-established as 20 nucleotides in Spy and the Sth systems, though a longer spacer sequence in a guide may provide increased efficiency in Cas9 targeting and reduced off-targeting.

The biological function of the ldrRNA is unknown^28^, and this is the first broad investigation into the expression patterns of the ldrRNA in multiple II-A systems. We hypothesize this RNA might provide a ruler-anchor mechanism for determining how crRNAs are processed due to its strict size conservation that matches the crRNAs processing boundaries. Additionally, the similarity between the ldrRNA and crRNA may suggest an alternative role for the ldrRNA priming Cas9 for adaptation or crRNA loading. We observed crRNAs expression across the array consistent with previous reports of expression trends in that expression is highest at the 5′ end of array^28,50^. The first crRNAs may be more stable because they are transcribed first, making them more available for tracrRNA-binding and protection by Cas9 and providing immunity against the most recently seen MGEs (Figs 4, S1).

Lactobacilli Cas9 are known to utilize tracrRNAs with unique sequences and structures; the diversity in these RNAs suggest each individual RNA is likely not compatible with Cas9s from other systems^12,36^. The lock-and-key specificity of tracrRNAs with Cas9s opens the door for multiplexing potential and concurrent use of different systems simultaneously for genome editing. Additionally, through understanding the native processing sites on tracrRNAs and crRNAs, minimal guide sequences can be used to develop single guide RNAs (sgRNAs) from these sequences (Figs 5, S5).

We were able to demonstrate that five different CRISPR-Cas systems have endogenous interference activity, with a range of interference efficiencies. The differences in PAM sequences suggest there is an entire spectrum of endogenous PAMs that can be used with different Cas9s. The range in ability to target and cleave could be a result of an imperfect PAM, differences in crRNA expression activity, differences in the background ability of the organisms to take-up plasmid DNA, or true differences in the targeting activity of each Cas9. With this assay we cannot compare Cas9 activity between organisms due to the design of the experiments meant to characterize endogenous activity of systems; however, interference levels within an organism can be compared to determine activity of each Cas9 with different PAMs.

This PAM flexibility may allow Cas9 to recognize sequences on rapidly mutating phages while also providing circumstantial evidence for a native mechanism of primed acquisition^7,15,27^. If Cas9 is able to flexibly target minor PAM mutants, it may bind the target long enough to acquire a new spacer from the invader. When defining what the true PAM is for each Cas9, it is important to consider there may be a difference between the sequences that allow for spacer acquisition and sequences that permit Cas9 recognition and binding^7,15,17,27,51^. When predicting the PAM using native protospacer sequences, we infer the sequence that Cas9 recognizes during the adaptation stage of CRISPR-Cas immunity; this prediction is likely more stringent than the total recognition space during the interference stage. When determining the PAM through depletion assays, broader flexibility is seen in PAM sequences which may be an evolutionary advantage during immunity as phages and other MGEs are known to rapidly evolve^7,15,52^.

It should be noted that the constructs with imperfect PAMs did occasionally show interference; this is likely due to PAM flexibility and the ability of Cas9 to promiscuously, though less effectively, recognize non-canonical or sub-ideal PAMs (Figs 6, S6). There were also instances where the predicted PAM was likely not the optimal PAM as many colonies were able to escape CRISPR targeting (Fig. S6). Escapees can point to several issues with CRISPR activity. The Cas9 protein may not be fully active and cannot fully eliminate all targets. Another possibility includes potential biases inherent to target sequences that affect the ability of Cas9 to interfere.

Once all components required for Cas9 functionality had been determined, we chose one system to develop into single guide RNA targeting technology. Interestingly, the ability to increase guide efficiency through mutagenesis seen here is contradictory to the Spy sgRNA data presented in Briner *et al*.^12^, and may be specific to lactobacilli or *L*. *gasseri*. This is the first investigation of perturbations allowable to double stemmed nexus tracrRNAs; modulation of Cas9 activity through mutations to the double stemmed nexus may be a function unique to these structures. Additionally, this is the first experiment to express an engineered single guide RNA and achieve self-targeting with an endogenous Cas9; previous approaches have relied on heterologous Cas machinery and engineered repeat-spacer arrays. This research opens the door to perform genome editing or targeted killing in bacteria containing native Cas9s with engineered sgRNAs.

Overall, here we present evidence of activity in the expression and interference phases of CRISPR immunity and circumstantial evidence for active acquisition in lactobacilli. Through investigation of the genetic diversity of CRISPR-Cas systems in hosts were they are naturally enriched, we found five potentially orthogonal systems that contain divergent Cas1s, Cas9s, ldrRNAs, CRISPR repeats, tracrRNAs, and PAMs. Insights into the transcriptional boundaries of the crRNAs and tracrRNAs during the expression stage, allowed us to successfully design a single guide RNA in *L*. *gasseri* that is able to mimic the native crRNA:tracrRNA duplex and have potentially designed guides that Cas9 can utilize better than the wildtype guides. We explored the native targets of CRISPR-Cas spacers to determine not only what predators attack lactobacilli, but were also able to infer what PAM sequence each Cas9 likely targets. Through plasmid interference assays, we confirmed the relative efficiency of each PAM and noticed a trend of flexible PAM targeting that may have implications both for the bacterial adaptive immunity and for genome editing applications of Cas9. In the literature it has been suggested that most CRISPR-Cas systems are not active or have low targeting activity against DNA^37,53^, but this does not appear to be the case with lactobacilli. The diversity of spacer sequences suggests lactobacilli live in a competitive environment under high phage pressure; likely due to the constant threat from invading DNA, CRISPR-Cas systems in lactobacilli need to be constitutively active and ready for defense.

The popularity of CRISPR-Cas systems exploded when Cas9 was first used as a genome-editing tool^54^. Through characterization of all three stages of CRISPR-Cas interference in Type II systems, we were able to develop the basic information necessary to develop potential new genome-editing tools that can be used both natively in bacteria and heterologously in eukaryotic systems. The systems we investigated here cluster into five consistent phylogenetic groups based on Cas1 sequence, Cas9 sequence, ldrRNA and crRNA sequence and length, tracrRNA sequence and structure, and PAM recognition sequence. Future studies will likely show these separate phylogenetic groups are orthogonal systems that contain independent machinery not capable of cross-talk and can be used to multiplex systems for genome editing. By characterizing the native functions of CRISPR-Cas machinery in their hosts, we are able to expand the Cas9 toolbox. The tools created from these systems will be capable of targeting a broader range of sequences due to novel PAM sequences, enabling more precise targeting, and can be used concurrently to multiplex with different Cas9s due to novel sgRNAs.

## Methods

### *In silico* analyses

1,262 *Lactobacillus* genomes were downloaded from NCBI (Table S1). CRISPR-Cas content was detected using the CRISPRdisco pipeline^55^. The core genome tree was generated using the proteins identified by Sun *et al*., 2015 and aligned used the CLC Genomics ® Workbench. The tree was generated with 100 bootstrap replications in CLC Genomics. The metadata was added to the tree with the results of our CRISPR-Cas annotations.

Protein sequences for the universal Cas1 protein and Type II signature protein, Cas9, were aligned using MUSCLE^56^. Neighbor-joining trees with 100 bootstrap replications were generated using MEGA6^57^; the Cas1 tree was rooted on the Type I-Type II CRISPR-Cas system split, while the Cas9 tree was rooted on the Type II-C branch containing *Neisseria meningitidis* and *Lactobacillus coryniformis*. The highly investigated Cas9 proteins from *Streptococcus pyogenes*, *Streptococcus thermophilus*, *Staphylococcus aureus*, and *N*. *meningitidis* were included in the analysis to demonstrate the diversity in the Cas9 dataset. A smaller subset of Cas9s from all the systems identified were selected for further characterization based diversity throughout the Cas9 space and uniqueness within the group.

Using the alignment of the Cas9 proteins, the protein motifs as identified by Nishimasu *et al*.^58^ for Spy Cas9 and Ran *et al*.^37^ for Sau Cas9 were mapped onto the selected subset of Cas9 proteins.

To identify native protospacer targets encoded by the CRISPR arrays, spacers were BLASTed against publically available data including the nr/nt, HTGS, WGS, and SRA databases (Table S2). Positive hits were defined as covering at least 80% of the spacer length with 90% or higher sequence identity. The 10 nucleotide flanking regions on the 5′ and 3′ ends of the protospacer sequences were aligned by hand and submitted to WebLogo^59^ for sequence motif identification.

### Plasmid generation with inserts

Interference plasmids were generated to test activity of CRISPR-Cas systems using native machinery *in vivo*. A protospacer sequence was selected for each organism by selecting a spacer that exhibited a highly expressed crRNA. PAM mutants were designed to test flexibility and spacing of the Cas recognition machinery. Double stranded inserts were generated by annealing extended oligos containing the protospacer, PAM, and *BamH*I/*Sac*I or *Hind*III/*Spe*I restriction sites. Plasmids were heat shocked into chemically competent *Escherichia coli* D10 or GM1829 cells and plated on selective media containing erythromycin and IPTG/Xgal (Thermo-Fischer). Positive clones were grown in overnight shaking cultures and plasmids were extracted using the QIAGEN Spin MiniPrep kit. The PAM and protospacer sequences were confirmed via Sanger sequencing at the NC State Genomic Science Lab (Raleigh, NC). Plasmids were quantified using a NanoDrop 2000c. Oligos used to generate these plasmids can be found in Table S3.

### Plasmid interference assay

Transformations were optimized for *Lactobacillus casei*, *Lactobacillus rhamnosus*, *Lactobacillus gasseri*, *Lactobacillus jensenii*, and *Lactobacillus pentosus*. Overnight cultures were inoculated into 100 mL of Man-de Rossa-Sharpe (MRS) broth with or without 2% glycine at an OD of 0.05 at 600 nm. Cultures were grown to OD 0.50, with some species receiving ampicillin at a final concentration of 10 ug/mL. Cells were pelleted by centrifugation at 5,000 × g for 15 minutes. Some cultures received a lithium acetate [7 mM phosphate buffer, pH 7.4, 600 mM sucrose, 100 mM lithium acetate, 10 mM dithiothreitol] incubation for 30 minutes and spinning at 4,500 × g for 15 minutes. Pellets were resuspended in 50 mL of 3.5X Sucrose Magnesium Electroporation Buffer (SMEB) buffer containing 7 nM phosphate buffer, pH 7.4, 952 mM sucrose, 3.5 mM MgCl_2_. The cultures were centrifuged at 4,000 × g for 15 minutes, resuspended in 25 mL 3.5X SMEB, centrifuged at 4,000 × g for 20 minutes, and resuspended in a final 1 mL of 3.5X SMEB. 100 uL of competent cells were added to 400 ng of plasmid and pipetted into a pre-chilled 2 mm gap electroporation cuvette. The cultures were electroporated at a constant voltage of 2.5 kV. Post electroporation, the cells were immediately added to 900 uL of pre-warmed MRS with 1%v/v recovery buffer [2 M sucrose, 20 mM CaCl_2_, 200 mM mgCl_2_] and recovered overnight. Cells were plated on MRS agar containing erythromycin and grown anaerobically for two to five days. Colonies were counted to determine interference capabilities of Cas9 with the different PAM variants. Standard error was calculated based on three replications.

### RNA-Seq

Cultures were grown to mid-log phase, harvested by centrifugation, and lysed via bead-beating in Trizol (Life Technologies, Carlsbad, CA) with 0.5 mm glass beads (MO BIO Laboratories, Carlsbad, CA). RNA was purified from the lysate using the Direct-zol RNA Miniprep Kit with in column DNase digestion (Zymo Research, Irvine, CA). Total RNA was submitted to the University of Illinois Roy J. Carver Biotechnology Center High-Throughput Sequencing and Genotyping Unit, and smRNA libraries were prepared with the NextFlex Small RNA-Seq Library Prep kit V2 (Bio Scientific, Austin, TX) for size-selected fragments 17 to 200 nt in length. The libraries were sequenced in a single lane of Illumina HiSeq. 2500 with a read length of 180 nt. Data was received de-multiplexed and uploaded into Geneious® for adapter removal followed by quality trimming to an error probability limit of 0.001, filtering to exclude reads <15 nt, and mapping to the reference genome for each species using Bowtie2^60^. Box plots were generated with the statistical program R.

### Self-targeting assay

Synthetic single guide RNAs (sgRNA) were designed for *L*. *gasseri* based on the RNA-Seq confirmed boundaries for the tracrRNA and crRNAs. A protospacer sequence flanked by the PAM 5′-cTAAC-3′ in the FruK was selected as the target for a chromosomal self-targeting assay. The corresponding spacer sequence was designed in the guide RNA. A highly expressed promoter for the *tuf* gene was cloned in front of the sgRNA. Using the transformation protocol for *L*. *gasseri* in the plasmid interference assays, plasmids containing the promoter and single guide were transformed into the cells. Overnight recovered cells were plated on minimal MRS containing 10% fructose, 3 ug/ml erythromycin, and bromocresol purple to assess the ability of the transformants to still metabolize fructose.

### Data availability

The BioProject ID for this experiment is PRJNA400806. The raw small RNA data can be reached using the following SRA Accession Numbers: SRR5997381-SRR5997390.

## Electronic supplementary material

Supplemental Figures

CRISPR-Cas systems in lactobacilli

Protospacer hits/PAM search

Oligos, vectors, and strains used in this study

## References

[1] Barrangou R (2007). CRISPR provides acquired resistance against viruses in prokaryotes. Science.

[2] Brouns SJ (2008). Small CRISPR RNAs guide antiviral defense in prokaryotes. Science.

[3] Marraffini LA, Sontheimer EJ (2008). CRISPR interference limits horizontal gene transfer in staphylococci by targeting DNA. Science.

[4] Hille, F. & Charpentier, E. CRISPR-Cas: biology, mechanisms and relevance. *Philos Trans R Soc Lond B Biol Sci* **371**, 10.1098/rstb.2015.0496 (2016). 10.1098/rstb.2015.0496 PMC5052741 27672148

[5] Barrangou R (2015). The roles of CRISPR-Cas systems in adaptive immunity and beyond. Curr Opin Immunol.

[6] Wei Y, Terns RM, Terns MP (2015). Cas9 function and host genome sampling in Type II-A CRISPR-Cas adaptation. Genes Dev.

[7] Paez-Espino D (2013). Strong bias in the bacterial CRISPR elements that confer immunity to phage. Nat Commun.

[8] Arslan Z, Hermanns V, Wurm R, Wagner R, Pul U (2014). Detection and characterization of spacer integration intermediates in type I-E CRISPR-Cas system. Nucleic Acids Res.

[9] Nuñez, J. K. *et al*. Cas1–Cas2 complex formation mediates spacer acquisition during CRISPR–Cas adaptive immunity. *Nat Struct Mol Biol* **21**, 528–534, 10.1038/nsmb.2820, http://www.nature.com/nsmb/journal/v21/n6/abs/nsmb.2820.html#supplementary-information (2014). 10.1038/nsmb.2820 PMC4075942 24793649

[10] Gasiunas G, Barrangou R, Horvath P, Siksnys V (2012). Cas9-crRNA ribonucleoprotein complex mediates specific DNA cleavage for adaptive immunity in bacteria. Proc Natl Acad Sci USA.

[11] Karvelis T (2013). crRNA and tracrRNA guide Cas9-mediated DNA interference in Streptococcus thermophilus. RNA biology.

[12] Briner AE (2014). Guide RNA functional modules direct Cas9 activity and orthogonality. Molecular cell.

[13] Sapranauskas R (2011). The Streptococcus thermophilus CRISPR/Cas system provides immunity in Escherichia coli. Nucleic acids research.

[14] Horvath P (2008). Diversity, activity, and evolution of CRISPR loci in Streptococcus thermophilus. J Bacteriol.

[15] Deveau H (2008). Phage response to CRISPR-encoded resistance in Streptococcus thermophilus. Journal of bacteriology.

[16] Marraffini LA, Sontheimer EJ (2010). Self versus non-self discrimination during CRISPR RNA-directed immunity. Nature.

[17] Mojica FJ, Diez-Villasenor C, Garcia-Martinez J, Almendros C (2009). Short motif sequences determine the targets of the prokaryotic CRISPR defence system. Microbiology.

[18] Grissa I, Vergnaud G, Pourcel C (2007). The CRISPRdb database and tools to display CRISPRs and to generate dictionaries of spacers and repeats. BMC Bioinformatics.

[19] Makarova KS (2015). An updated evolutionary classification of CRISPR-Cas systems. Nat Rev Microbiol.

[20] Shmakov S (2017). Diversity and evolution of class 2 CRISPR-Cas systems. Nat Rev Microbiol.

[21] Burstein D (2017). New CRISPR-Cas systems from uncultivated microbes. Nature.

[22] Jinek M (2012). A programmable dual-RNA–guided DNA endonuclease in adaptive bacterial immunity. Science.

[23] Cong L (2013). Multiplex genome engineering using CRISPR/Cas systems. Science.

[24] Mali P (2013). RNA-guided human genome engineering via Cas9. Science.

[25] Sun Z (2015). Expanding the biotechnology potential of lactobacilli through comparative genomics of 213 strains and associated genera. Nat Commun.

[26] Horvath P (2009). Comparative analysis of CRISPR loci in lactic acid bacteria genomes. Int J Food Microbiol.

[27] Paez-Espino, D. *et al*. CRISPR immunity drives rapid phage genome evolution in Streptococcus thermophilus. *MBio* **6**, 10.1128/mBio.00262-15 (2015). 10.1128/mBio.00262-15 PMC4453577 25900652

[28] Wei Y, Chesne MT, Terns RM, Terns MP (2015). Sequences spanning the leader-repeat junction mediate CRISPR adaptation to phage in Streptococcus thermophilus. Nucleic Acids Res.

[29] Held NL, Herrera A, Whitaker RJ (2013). Reassortment of CRISPR repeat-spacer loci in Sulfolobus islandicus. Environ Microbiol.

[30] Deng L, Garrett RA, Shah SA, Peng X, She Q (2013). A novel interference mechanism by a type IIIB CRISPR-Cmr module in Sulfolobus. Mol Microbiol.

[31] Haurwitz RE, Jinek M, Wiedenheft B, Zhou K, Doudna JA (2010). Sequence- and structure-specific RNA processing by a CRISPR endonuclease. Science.

[32] Wiedenheft B (2009). Structural basis for DNase activity of a conserved protein implicated in CRISPR-mediated genome defense. Structure.

[33] Ivancic-Bace I, Cass SD, Wearne SJ, Bolt EL (2015). Different genome stability proteins underpin primed and naive adaptation in E. coli CRISPR-Cas immunity. Nucleic Acids Res.

[34] Toms A, Barrangou R (2017). On the global CRISPR array behavior in class I systems. Biol Direct.

[35] Smargon AA (2017). Cas13b Is a Type VI-B CRISPR-Associated RNA-Guided RNase Differentially Regulated by Accessory Proteins Csx27 and Csx28. Mol Cell.

[36] Esvelt, K. M. *et al*. Orthogonal Cas9 proteins for RNA-guided gene regulation and editing. *Nat Meth* **10**, 1116–1121, 10.1038/nmeth.2681, http://www.nature.com/nmeth/journal/v10/n11/abs/nmeth.2681.html#supplementary-information (2013). 10.1038/nmeth.2681 PMC3844869 24076762

[37] Ran, F. A. *et al*. *In vivo* genome editing using Staphylococcus aureus Cas9. *Nature* **520**, 186–191, 10.1038/nature14299, http://www.nature.com/nature/journal/v520/n7546/abs/nature14299.html#supplementary-information (2015). 10.1038/nature14299 PMC4393360 25830891

[38] Deltcheva E (2011). CRISPR RNA maturation by trans-encoded small RNA and host factor RNase III. Nature.

[39] Pertzev AV, Nicholson AW (2006). Characterization of RNA sequence determinants and antideterminants of processing reactivity for a minimal substrate of Escherichia coli ribonuclease III. Nucleic Acids Res.

[40] Sanozky-Dawes R, Selle K, O’Flaherty S, Klaenhammer T, Barrangou R (2015). Occurrence and activity of a type II CRISPR-Cas system in Lactobacillus gasseri. Microbiology.

[41] Chaudhary, K., Chattopadhyay, A. & Pratap, D. Anti-CRISPR proteins: Counterattack of phages on bacterial defense (CRISPR/Cas) system. *J Cell Physiol*, 10.1002/jcp.25877 (2017). 10.1002/jcp.25877 28247934

[42] Hynes, A. P. *et al*. An anti-CRISPR from a virulent streptococcal phage inhibits Streptococcus pyogenes Cas9. *Nat Microbiol*, 10.1038/s41564-017-0004-7 (2017). 10.1038/s41564-017-0004-7 28785032

[43] Pawluk A (2016). Naturally Occurring Off-Switches for CRISPR-Cas9. Cell.

[44] Li M, Wang R, Xiang H (2014). Haloarcula hispanica CRISPR authenticates PAM of a target sequence to prime discriminative adaptation. Nucleic Acids Res.

[45] Li M, Wang R, Zhao D, Xiang H (2014). Adaptation of the Haloarcula hispanica CRISPR-Cas system to a purified virus strictly requires a priming process. Nucleic Acids Res.

[46] Levy A (2015). CRISPR adaptation biases explain preference for acquisition of foreign DNA. Nature.

[47] Fagerlund RD (2017). Spacer capture and integration by a type I-F Cas1-Cas2-3 CRISPR adaptation complex. Proc Natl Acad Sci USA.

[48] Staals RH (2016). Interference-driven spacer acquisition is dominant over naive and primed adaptation in a native CRISPR-Cas system. Nat Commun.

[49] Fineran PC (2014). Degenerate target sites mediate rapid primed CRISPR adaptation. Proc Natl Acad Sci USA.

[50] McGinn J, Marraffini LA (2016). CRISPR-Cas Systems Optimize Their Immune Response by Specifying the Site of Spacer Integration. Mol Cell.

[51] Leenay RT, Beisel CL (2017). Deciphering, Communicating, and Engineering the CRISPR PAM. J Mol Biol.

[52] Leenay RT (2016). Identifying and Visualizing Functional PAM Diversity across CRISPR-Cas Systems. Mol Cell.

[53] Kleinstiver BP (2015). Engineered CRISPR-Cas9 nucleases with altered PAM specificities. Nature.

[54] Barrangou R, Doudna JA (2016). Applications of CRISPR technologies in research and beyond. Nat Biotechnol.

[55] Crawley, A. B., Henriksen, J. R. & Barrangou, R. CRISPRdisco: an automated pipeline for the discovery and analysis of CRISPR-Cas systems. *The CRISPR Journal* **1**, epub ahead of print. (2018). 10.1089/crispr.2017.0022 PMC6636876 31021201

[56] Edgar RC (2004). MUSCLE: multiple sequence alignment with high accuracy and high throughput. Nucleic Acids Res.

[57] Tamura K, Stecher G, Peterson D, Filipski A, Kumar S (2013). MEGA6: Molecular Evolutionary Genetics Analysis version 6.0. Mol Biol Evol.

[58] Nishimasu H (2014). Crystal structure of Cas9 in complex with guide RNA and target DNA. Cell.

[59] Crooks GE, Hon G, Chandonia JM, Brenner SE (2004). WebLogo: a sequence logo generator. Genome Res.

[60] Langdon WB (2015). Performance of genetic programming optimised Bowtie2 on genome comparison and analytic testing (GCAT) benchmarks. BioData Min.

