# Supplementary material for: Characterizing the activity of abundant, diverse and active CRISPR-Cas systems in lactobacilli
Source: Sci Rep. 2018 Aug 1;8:11544. doi: 10.1038/s41598-018-29746-3 (PMC6070500; doi:10.1038/s41598-018-29746-3)
Supplement: Supplementary file 1 — Supplemental Figures [file 41598_2018_29746_MOESM1_ESM.pdf]

Characterizing the activity of abundant, diverse and active CRISPR-Cas systems in lactobacilli

Alexandra B Crawley, Emily D Henriksen, Emily Stout, Katelyn Brandt, Rodolphe Barrangou

Supp Fig 1 | Transcription profiles for entire repeat-spacer arrays

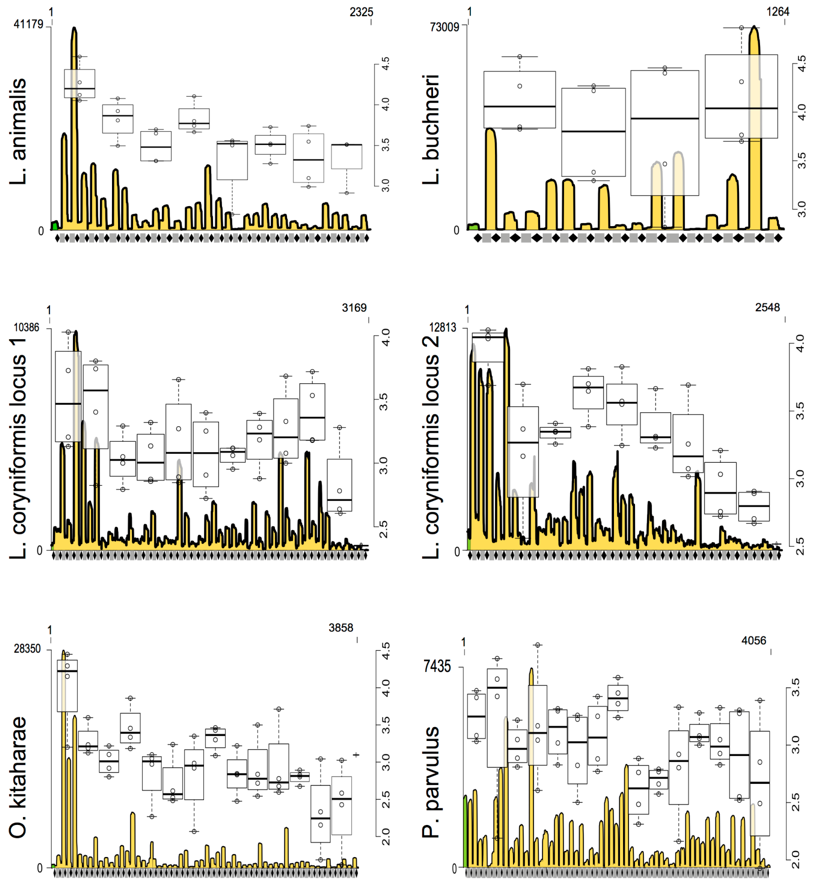

Expression boundaries of the entire repeat-spacer arrays are shown for additional species. The grey boxes represent CRISPR spacers; the black boxes represent CRISPR repeats. The box plots show summary statistics on the expression profiles of the arrays.

Supp Fig 2 | Transcription profiles for crRNAs

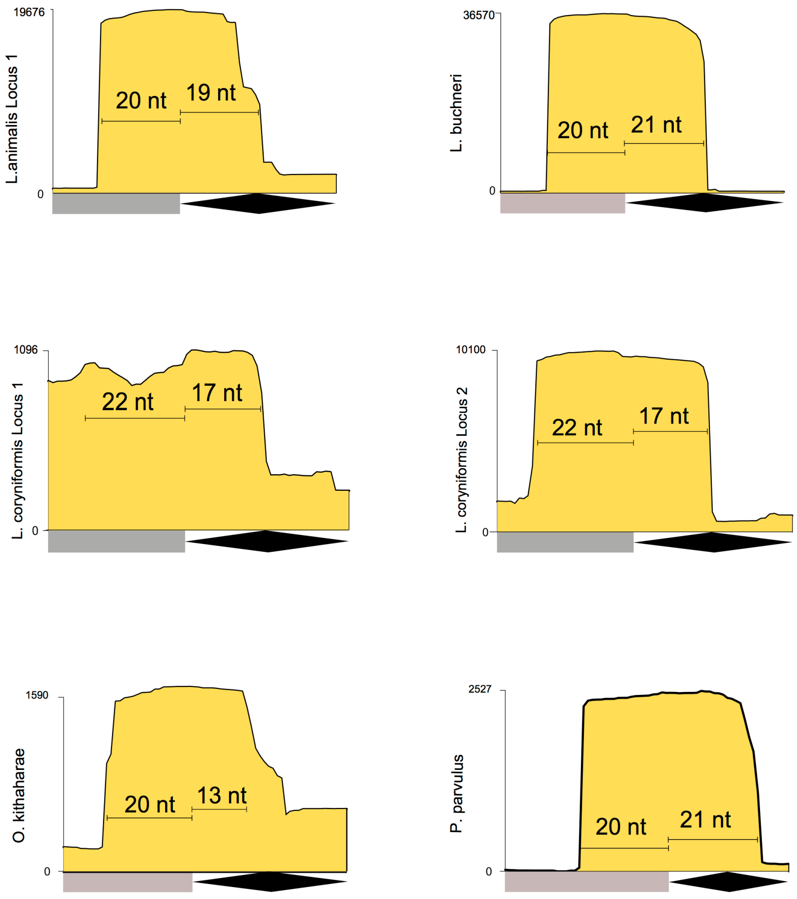

Expression boundaries of most highly expressed crRNA in array. The grey boxes represent CRISPR spacers; the black boxes represent CRISPR repeats.

Supp Fig 3 | Transcription profiles for ldrRNAs

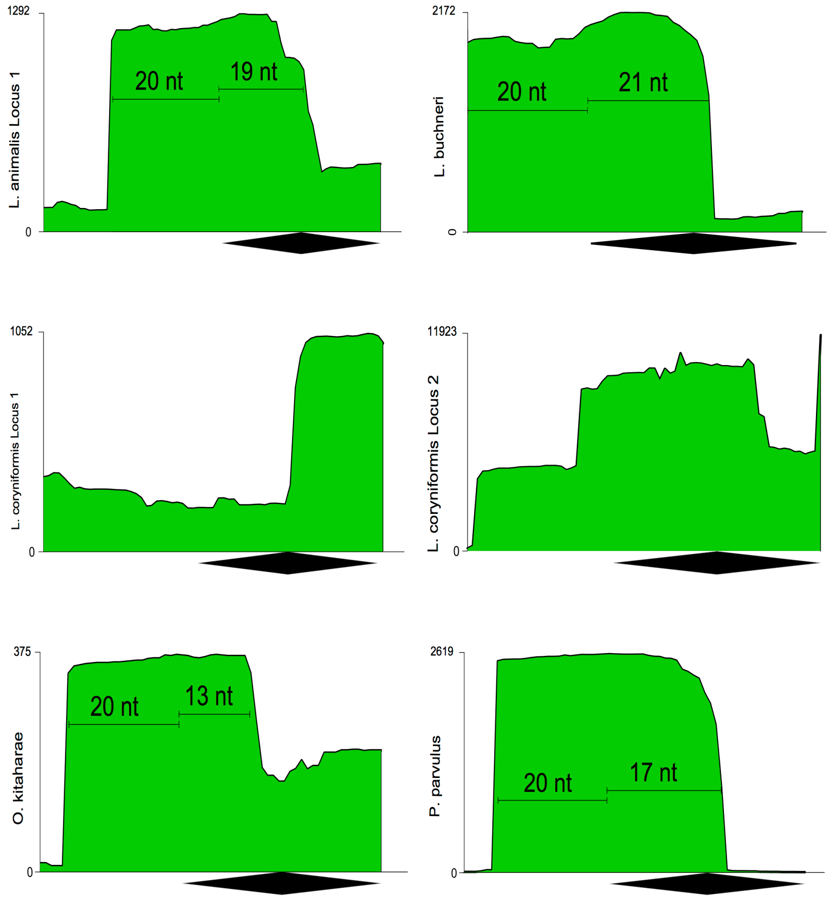

Expression boundaries of ldrRNA across the first CRISPR repeat (black diamond).

Supp Fig 4 | Transcription profiles for tracrRNAs

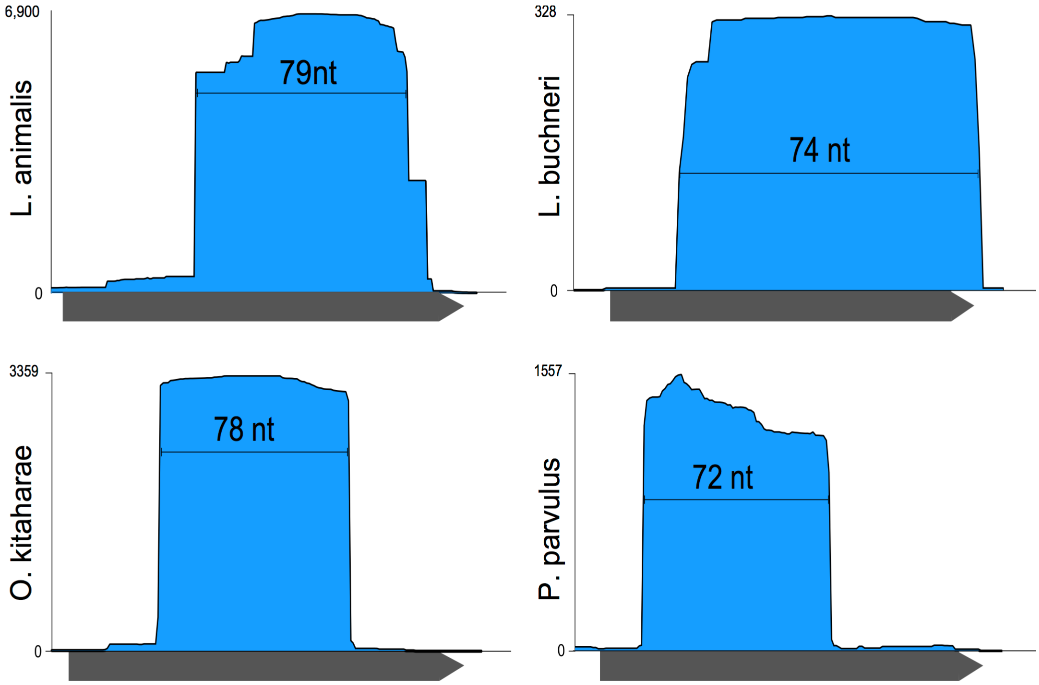

Expression boundaries spanning predicted tracrRNA sequences (gray arrow). No tracrRNA was detected for L coryniformis which is a II-C system.

Supp Fig 5 | Extended endogenous crRNA:tracrRNA duplexes

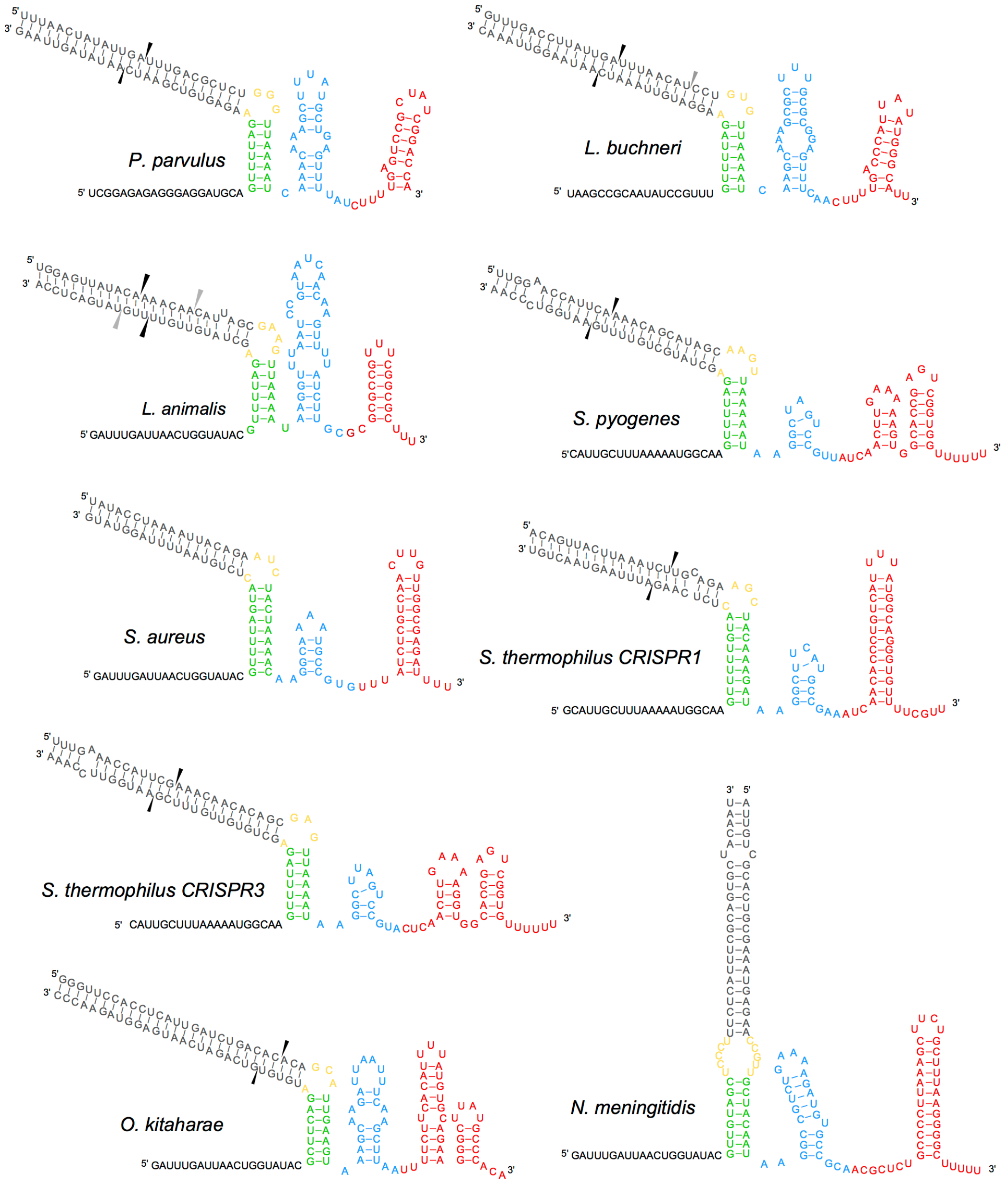

Predicted secondary structure of crRNA:tracrRNA duplex investigated in this study and previously established. Sequences colored by guide module: green (lower stem), yellow (bulge), blue (nexus), red (terminal hairpins).

Supp Fig 6 | Extended interference assays for all PAM plasmid interference assays

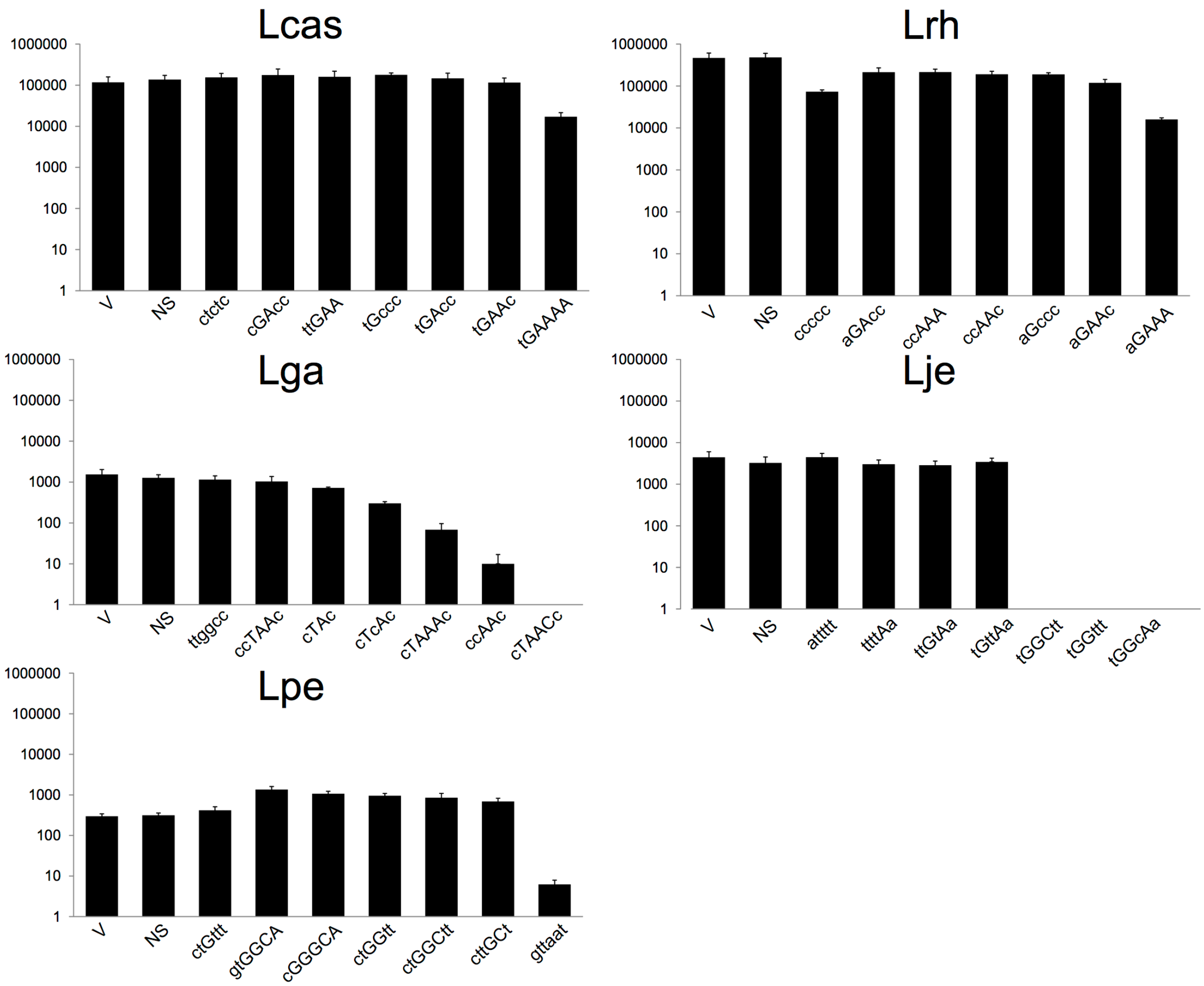

Plasmid interference assays were used to test the ability of each Cas9 to recognize and cleave plasmid DNA. Several constructs were used to determine plasmid interference, including empty vector (V), no spacer target (NS), no PAM targets (NP), and vectors that contained both targets and potential PAM sequences. The y-axis shows the log number of transformants recovered with each PAM mutation. Error bars are based on three independent replicates.

Supp Fig 7 | Experimental Procedures

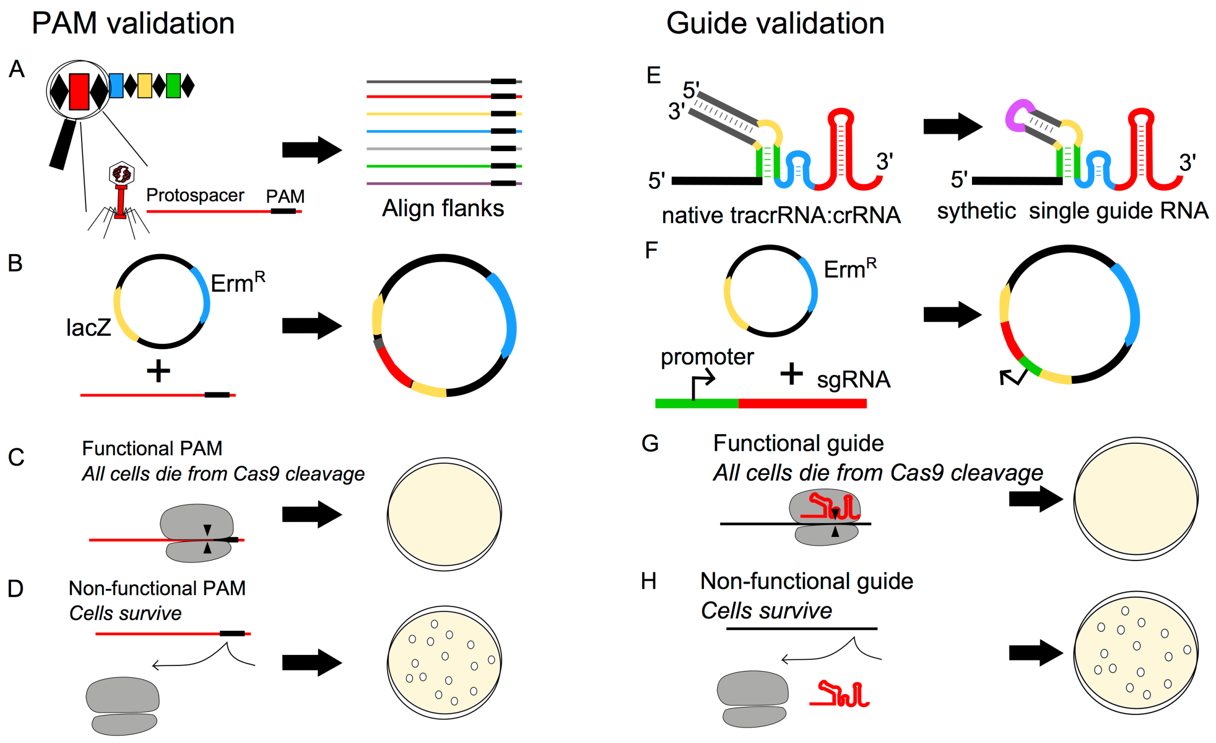

(A) Each spacer is used to determine its original protospacer sequence. The protospacer flanks are aligned to determine the PAM sequence. (B) Interference plasmids are generated by cloning a spacer and PAM sequence into a plasmid with a selectable marker. (C) If the PAM is correct and the CRISPR system is functional, the Cas9 will cleave the plasmid and no cells will be recovered on the selective media. (D) If the PAM in unrecognizable by Cas9, the protein will be unable to bind and cleave, thus allowing the plasmid to replicate and confer antibiotic resistance.
